# Examining the Role of Psychological Symptoms and Safety Climate in Shaping Safety Behaviors Among Construction Workers

**DOI:** 10.3390/bs15010066

**Published:** 2025-01-13

**Authors:** Na Guo, Yong Liu, Shiwang Yu, Bo Xia, Weiyi Cong

**Affiliations:** 1 School of Economic Management, Nanjing Vocational University of Industry Technology, Nanjing 210023, China; guon@niit.edu.cn; 2 School of Civil Engineering and Architecture, Zhejiang Sci-Tech University, Hangzhou 310018, China; jhly1007@zstu.edu.cn (Y.L.); wycong@zstu.edu.cn (W.C.); 3 School of Architecture and Built Environment, Faculty of Engineering, Queensland University of Technology, 2 George Street, Brisbane, QLD 4120, Australia; paul.xia@qut.edu.au

**Keywords:** construction worker, psychological symptoms, safety climate, safety behaviors

## Abstract

Construction workers are often subjected to strenuous manual labor, poor working conditions, and prolonged separation from family, leading to psychological symptoms such as stress, depression, and anxiety. These psychological factors, combined with safety climate elements like risk perception and safety attitude, significantly influence workers’ safety behaviors, including risk-taking, safety compliance, and safety participation. To address these issues, this study investigates the interplay between psychological symptoms, safety climate, and safety behaviors among construction workers in Mainland China. Data were collected through a survey of 160 construction workers, with results analyzed using correlation and regression techniques. The findings reveal that: (1) risky behavior is primarily driven by anxiety and risk perception; (2) safety participation is influenced by safety attitudes and levels of depression; and (3) safety compliance is affected by risk perception, safety attitude, stress, and depression. Interestingly, an inverse relationship is observed between depression and both risky behavior and safety compliance. These results underscore the importance of addressing psychological well-being to improve safety outcomes. Practical implications include providing psychological counseling, stress management training, and improving social connections for workers, such as facilitating regular video calls with family members or offering travel reimbursements.

## 1. Introduction

The construction industry is a cornerstone of national economies and one of the largest employers in many countries (Asare et al., 2022). In Mainland China, rapid economic development has fueled significant growth in the construction sector, which now employs an estimated 300 million construction workers (CWs) (National Bureau of Statistics, 2023). Due to the demanding nature of construction work, CWs often endure physically intensive tasks to meet daily job requirements, leading to various physical health symptoms (Yasmeen et al., 2020). Compounding this, a significant proportion of CWs in Mainland China are rural migrant workers who live on-site in dormitories with substandard living conditions (Zhong et al., 2018). These workers frequently face long separations from their families, combined with an unfamiliar work environment, contributing to discomfort and exacerbating psychological challenges such as anxiety and depression. Based on Dai’s (Dai, 2023) research, 52.43%, 51.31%, and 23.60% of practitioners experienced varying levels of depression, anxiety, and stress, respectively.

The construction industry in Mainland China also faces a disproportionate share of workplace accidents (Zhang et al., 2019). CWs are routinely exposed to hazardous conditions, and accidents—both fatal and non-fatal—remain a pervasive threat. Enhanced safety behaviors among CWs, including adherence to safety protocols and active participation in safety practices, are critical to reducing accident rates (Mahmoud et al., 2021). Accidents not only lead to severe human costs, including injuries and fatalities, but also impose substantial financial burdens on construction companies through compensation payments, project delays, and fines (Lee et al., 2021). Such incidents can tarnish a company’s reputation, diminishing its competitiveness in future tenders.

Empirical evidence underscores the importance of positive safety behaviors in mitigating accident risks (Xia et al., 2020). CWs who exhibit safe behaviors are significantly less likely to encounter workplace accidents (Zhang et al., 2023). Prior research has identified factors such as safety attitudes and risk perception—commonly encompassed under the term “safety climate”—as influential determinants of CW behaviors (Shea et al., 2021). Creating a positive safety climate helps to encourage construction workers to implement safe and compliant behaviors during construction operations. On the other hand, due to the long hours and high intensity of work, the psychological state of construction workers is often in a negative and unhealthy situation. There are a large number of studies showing that construction workers suffer from severe psychological stress that can even negatively affect their safety behaviors. Stress has been recognized as a critical factor affecting safety behaviors (Pooladvand & Hasanzadeh, 2023; Renecle et al., 2021). Existing studies have revealed the causal relationship between psychological stress among construction workers and their safety behaviors and safety performance (Leung et al., 2012; Liang et al., 2022). At the same time, a study has also shown that psychological stress among construction workers has a direct effect on the safety climate (Chen et al., 2017). However, there remains a notable gap in understanding the intricate interrelationships between CWs’ psychological symptoms, safety climate, and safety behaviors. This study seeks to address this research gap, providing a nuanced exploration of these interdependencies and offering insights to enhance the safety and well-being of CWs in the construction industry.

The contributions of this study are as follows: First, it systematically explores, for the first time, the interactions among three types of psychological symptoms (stress, anxiety, and depression) and their complex relationships with three types of safety behaviors (risky behaviors, safety compliance, and safety participation), revealing the multi-level mechanisms through which mental health influences safety behaviors. Second, it identifies the inverse effect of depression on risky behaviors and safety compliance through empirical data, offering a new perspective on the existing literature. Lastly, by integrating psychological symptoms, safety climate, and safety behaviors, this study develops a multidimensional analytical framework, providing a theoretical foundation for future research in related fields.

The reminder of this paper is structured as follows: Section 2 provides a literature review, formulates the conceptual model based on empirical evidence, and outlines the research methodology, detailing the population and sample, data collection, and analytical procedures. Subsequent sections present the empirical results, discuss the findings, and draw conclusions. Section 5 offers practical implications based on the study’s conclusions, while Section 6 addresses the study’s implications and proposes avenues for future research, aiming to enhance our understanding of the link between psychological well-being and safety performance in the construction industry.

## 2. Materials and Methods

### 2.1. Materials

#### 2.1.1. Psychological Symptoms

Construction workers (CWs) frequently perform physically demanding tasks in hazardous and adverse conditions, often under pressure to meet stringent deadlines (Ahmed et al., 2022). Their work, such as tying steel rods, laying formwork, and pouring concrete, involves prolonged manual labor, exposing them to substantial physical and psychological stressors (Koteng, 2022; Lingard, 2019) Research in the fields of occupational health and stress management consistently underscores the prevalence of psychological symptoms (PSs) among CWs, which adversely affect their well-being, productivity, and workplace safety (Sun et al., 2022).

Psychological symptoms are commonly categorized into three primary negative emotional states: depression, anxiety, and stress (Langdon & Sawang, 2018). Depression is characterized by persistent feelings of sadness, hopelessness, and emotional detachment, leading to diminished engagement in both professional and personal activities (Tavella & Parker, 2020). Depressed workers often experience lower perceptions of safety, reduced safety performance, and higher healthcare costs, reflecting their vulnerability to workplace hazards (Black et al., 2019). Furthermore, depression impairs cognitive functions such as attention and decision-making, contributing to non-compliance with safety protocols and maladaptive coping strategies like anxious avoidance or behavioral disengagement (Ali & Dominic, 2024). These factors also influence their risk perception, potentially heightening exposure to accidents.

Anxiety, another critical psychological symptom, manifests as a state of excessive worry, nervousness, or unease, often accompanied by physical symptoms such as trembling, sweating, and panic attacks (Panday & Jha, 2025). The demanding work schedules and suboptimal working environments of CWs exacerbate anxiety levels, leading to reduced safety compliance and increased risk perception (Gao et al., 2020). Workers with high anxiety levels may exhibit negative attitudes toward safety practices and engage in riskier behaviors, amplifying the likelihood of accidents (Zong et al., 2025).

Stress, defined as a state of mental or emotional strain resulting from adverse or demanding circumstances, is particularly pervasive among CWs due to the nature of their work environment and heavy workload (Tijani et al., 2020). Chronic exposure to stress not only deteriorates physical and mental health but also impairs focus, judgment, and adherence to safety protocols (Lee et al., 2024). Prolonged stress may result in heightened emotional reactivity, further compromising personal and workplace safety (Stelnicki et al., 2021). Addressing stress through effective interventions is thus critical to ensuring the well-being and performance of CWs.

#### 2.1.2. Safety Climate

The safety climate reflects the collective perceptions of workers regarding safety practices, policies, and the overall safety culture within their workplace. It serves as a vital predictor of safety behavior, accident rates, and organizational safety performance (Syed-Yahya et al., 2022). While there is no universally accepted framework for measuring safety climate, it is often analyzed through dimensions such as risk perception and safety attitudes (Pandit et al., 2019).

Risk perception refers to an individual’s awareness and assessment of potential hazards, including the likelihood and severity of harm (Siegrist & Árvai, 2020). It is influenced by personal experiences, cultural background, and situational factors, making it a dynamic component of workplace safety (Marshall, 2020). Accurate risk perception is crucial for informed decision-making, as it enables workers to anticipate and mitigate potential dangers effectively (Leder et al., 2019). Conversely, underestimating risks can result in non-compliance with safety protocols and increased exposure to hazardous situations.

Safety attitudes represent workers’ beliefs, perceptions, and values regarding the importance of safety in their work environment (Oah et al., 2018). These attitudes shape their commitment to safety practices and their responses to safety challenges (Newaz et al., 2019). In high-risk industries like construction, fostering positive safety attitudes is essential for reducing accidents and enhancing compliance with safety standards (Kadher et al., 2024). Unlike innate traits, safety attitudes are malleable and can be influenced by workplace interventions, leadership styles, and psychological factors such as stress and anxiety (Kao et al., 2021). Organizations must therefore focus on cultivating a supportive safety culture that reinforces positive attitudes and addresses underlying psychological issues that may hinder safety compliance.

#### 2.1.3. Safety Behaviors

Safety behaviors encompass actions taken by workers to either adhere to or deviate from safety protocols, directly impacting workplace safety outcomes (Renecle et al., 2021). These behaviors are typically categorized into three types: risky behaviors, safety compliance, and safety participation (Derdowski & Mathisen, 2023; Peng & Chan, 2019; Sheedy & Griffin, 2018).

Risky behaviors involve actions that violate safety standards, such as neglecting to wear protective equipment, engaging in unsafe practices, or consuming alcohol or tobacco on-site (Han et al., 2022). These behaviors are often driven by individual traits such as sensation-seeking tendencies or by inaccurate risk perceptions, where workers underestimate the dangers of their actions (Sun et al., 2020). Reducing risky behaviors requires targeted interventions to address both individual and organizational factors contributing to such tendencies.

Risky behaviors, such as neglecting protective equipment or engaging in unsafe practices, violate safety standards and are often driven by individual traits like inaccurate risk perceptions, where workers underestimate the hazards of their actions (Han et al., 2022; Sun et al., 2020). Research highlights the pivotal role of safety attitudes and climate in mitigating such behaviors. Bhandari et al. (2021) demonstrated that a robust safety climate negatively correlates with risk tolerance and independently curtails risk-taking decisions, while Danso et al. (2023) linked risky behavior to safety incidents, stressing its persistence in unresolved safety dilemmas. Psychological factors are equally critical; Tixier et al. (2014) noted that workers with negative psychological states are more prone to risk-taking due to heightened risk perception, and Aulin et al. (2019) emphasized stress as a key driver of unsafe behaviors.

Safety compliance refers to workers’ adherence to established rules, regulations, and procedures designed to ensure a safe work environment (Hu et al., 2020). Compliance is particularly critical in the construction industry, where hazards are pervasive and potentially life-threatening. However, psychological symptoms such as stress, anxiety, and depression can significantly undermine workers’ ability to comply with safety regulations (Yan et al., 2023). Efforts to improve compliance should extend beyond punitive measures to include training programs, support systems, and organizational initiatives that address the root causes of non-compliance.

Safety participation denotes proactive involvement in promoting workplace safety, extending beyond individual responsibilities to include collaborative efforts to improve safety for all workers (Curcuruto et al., 2020). Unlike compliance, which focuses on rule adherence, participation emphasizes a voluntary, collective commitment to safety (Choi & Lee, 2022). Workers who actively engage in safety participation initiatives often contribute to a safer work environment, reducing accident rates and fostering a culture of shared responsibility (Homann et al., 2022). Encouraging participation requires cultivating an inclusive and empowering organizational culture that values worker input and prioritizes safety as a collective goal.

#### 2.1.4. The Conceptual Model

Based on the literature review and the conceptual framework, we propose the following hypotheses to examine the relationships among psychological symptoms (PSs), safety climate, and safety behaviors of construction workers (CWs). These hypotheses are grounded in the realities of construction work, including the high-stress environment, strenuous manual labor, and unique psychosocial dynamics of CWs.

Construction workers often face challenging work environments, including physical strain, long hours, and limited social interaction, which contribute to psychological distress. Depression, anxiety, and stress are closely linked, creating a cycle of mutual influence. Depression and anxiety often co-occur, with depressive symptoms increasing anxiety about job performance and future uncertainties. Similarly, chronic anxiety can worsen depression. Stress, caused by heavy workloads or workplace conflicts, can reduce psychological resilience, triggering depression and reinforcing stress. Finally, stress and anxiety are interconnected, with ongoing stress leading to chronic anxiety, which in turn amplifies emotional and physical stress. Based on these relationships, the following hypotheses are proposed:

**H_1.1_:**  *Depression has a mutual significant association with anxiety among CWs.*

**H_1.2_:**  *Depression has a mutual significant association with stress among CWs.*

**H_1.3_:**  *Anxiety has a mutual significant association with stress among CWs.*

In the construction industry, safety climate is crucial for workplace safety. Psychological symptoms like depression, anxiety, and stress can affect key elements of safety climate. Studies show that these symptoms can influence workers’ risk perception and safety attitudes. Depression may reduce risk awareness, making workers less likely to recognize hazards and more likely to disregard safety measures. Anxiety can increase risk perception but, if prolonged, may undermine trust in safety measures and negatively affect safety attitudes. Stress, often caused by heavy workloads or conflicts, can impair risk assessment and lead to negative views on safety regulations, seeing them as an added burden. Based on these relationships, the following hypotheses are proposed:

**H_2.1_:**  *Depression has a significant effect on the safety climate among CWs.*

**H_2.2_:**  *Anxiety has a significant effect on the safety climate among CWs.*

**H_2.3_:**  *Stress has a significant effect on the safety climate among CWs.*

Psychological symptoms such as depression, anxiety, and stress can directly affect construction workers’ safety behaviors, including risky behavior, safety compliance, and safety participation. Depression often leads to disengagement from safety measures, resulting in increased risky behaviors and reduced safety compliance and participation. Anxiety can heighten vigilance, potentially improving safety compliance, but may also lead to risky behavior due to impaired judgment, while reducing safety participation. Stress may cause workers to prioritize speed over safety, leading to more risky behavior and decreased safety compliance and participation. Thus, the study proposes the following hypotheses:

**H_3.1_:**  *Depression has a significant effect on safety behaviors among CWs.*

**H_3.2_:**  *Anxiety has a significant effect on safety behaviors among CWs.*

**H_3.3_:**  *Stress has a significant effect on safety behaviors among CWs.*

The safety climate, encompassing risk perception and safety attitude, plays a crucial role in shaping construction workers’ safety behaviors, including risky behavior, safety compliance, and safety participation. A strong risk perception—workers’ ability to identify and evaluate workplace hazards—discourages risky behavior while promoting safety compliance and participation. Similarly, a positive safety attitude—reflecting workers’ commitment to safety values and practices—enhances adherence to safety protocols and encourages proactive involvement in safety initiatives, reducing the likelihood of unsafe acts. Based on these dynamics, the following hypotheses are proposed:

**H_4.1_:**  *Risk perception directly reduces risky behavior while increasing safety compliance and participation.*

**H_4.2_:**  *Safety attitude directly reduces risky behavior while enhancing safety compliance and participation.*

**Figure 1:**
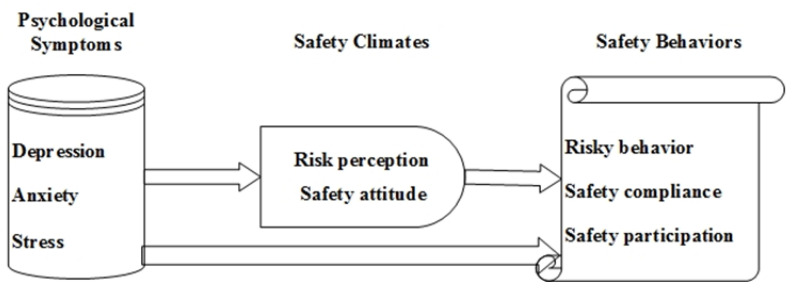
Conceptual model of PSs, safety climate, and safety behaviors for CWs.

The conceptual model illustrating these interconnections is presented in Figure 1.

### 2.2. Methods

#### 2.2.1. Survey Design

The background section gathered demographic and professional data, including gender, age, years of experience, education level, organization type, and job position. Psychological symptoms were assessed using a 15-item scale adapted from the Depression Anxiety Stress Scale (DASS), which measures three dimensions: depression, anxiety, and stress (Moussa et al., 2017). Each dimension was evaluated with five items, with responses rated on a 7-point Likert scale ranging from 1 (never happens/strongly disagree) to 7 (very frequently happens/strongly agree).

Safety climate was measured using two sub-scales, safety perception and safety attitude, based on established frameworks in the literature (see Table 1). Safety behaviors, encompassing risky behavior, safety compliance, and safety participation, were evaluated based on previous studies (see Table 1). For both safety climate and safety behavior items, participants rated statements on a 7-point Likert scale, where 1 represented “strongly disagree” and 7 signified “strongly agree”.

#### 2.2.2. Sample

Data were collected from construction workers (CWs) employed on construction sites in Shandong and Jiangsu Provinces between 2023 and 2024. To account for the varying educational backgrounds of participants, trained investigators provided detailed explanations during a briefing session to ensure understanding. A purposive sampling method was employed to select workers who met the following criteria: (1) proficiency in a skilled trade (e.g., brick mason, carpenter, concreter), and (2) current employment in mainstream construction sectors (e.g., main contractors, subcontractors, suppliers). To suit the respondents, who were Chinese workers, the questionnaires were translated into Chinese by the research team, ensuring consistency with the original English version.

Of the 253 questionnaires distributed via email, instant messaging platforms, and in-person interactions on-site, 186 responses were received, resulting in a response rate of 63.24%. After checking the received questionnaires according to the respondent inclusion criteria, 26 invalid responses were excluded from the final data analysis due to incompleteness. In total, 160 valid responses are incorporated into the final analysis, representing a response rate of 63.24%. The majority of the respondents are in the age group of 30–39 (36.8%), followed by 40–49 (31.6%) and over 50 (21.7%), while few respondents are identified in the age groups of 0–20 (1.6%) and 20–29 (8.3%). The majority of the respondents have worked in the construction industry for 6–10 years (32.5%), followed by 1–5 years (18.7%), 11–15 years (28.8%), 16–20 years (10%), with the few remaining CWs working in construction more than 20 years and less than 1 year. Of all respondents, 21.3% are primary school graduates, 43.8% have a junior high school education and 23.8% have a senior high school education, with only 6.3% having bachelor’s degrees. Most of the respondents work for sub-contractor firms, accounting for 41.3% of all respondents, with the remaining respondents working for main contractors (13.8%), suppliers (7.5%), and others (7.6%).

#### 2.2.3. Data Analysis

The data analysis followed a structured multi-step approach to ensure the reliability and validity of the results. Initially, factor analysis with varimax rotation (eigenvalue > 1) was employed to distill the dataset into meaningful constructs, adhering to a factor loading threshold of 0.6, which is appropriate given the sample size (Schreiber, 2021). Reliability analysis was subsequently conducted to evaluate the internal consistency of the constructs, using Cronbach’s alpha with a threshold value of 0.6 as the minimum criterion for acceptability (Reliability Analysis, 2018).

To explore the relationships among psychological symptoms (PSs), safety climate, and safety behaviors, Pearson correlation analysis was performed, offering insights into the strength and direction of associations among these variables (Baak et al., 2020). Finally, stepwise multiple regression analysis was applied to identify the interdependent relationships and the predictive strength of the independent variables on the dependent variables. This approach facilitated a nuanced understanding of the underlying dynamics among PSs, safety climate, and safety behaviors.

The Pearson correlation coefficient (r), ranging from −1 to 1, was used to represent perfect negative correlation, perfect positive correlation, and no linear correlation, respectively. A two-tailed t-test was conducted to determine the significance of these correlations, with the significance level set at α = 0.05 (Kim & Choi, 2021).

Stepwise multiple regression analyses were subsequently employed to evaluate the interdependence and predictive strength of the independent variables on the dependent variable. The regression coefficients (B) quantified the contribution of each independent variable, while their standard errors (S.E.) assessed the stability of these estimates (Montgomery et al., 2021). The significance of each independent variable was determined through t-values and corresponding *p*-values, using α = 0.05 as the threshold for significance. To address potential multicollinearity, the variance inflation factor (VIF) was calculated, with values below 10 indicating acceptable levels. The model’s overall explanatory power was assessed using the coefficient of determination (R^2^), where values closer to 1 signified stronger predictive accuracy (Okoye & Hosseini, 2024). Additionally, the model’s validity was confirmed through analysis of variance (ANOVA), which evaluated its overall significance.

## 3. Results

The items measuring psychological symptoms, safety climate, and safety behaviors were subjected to separate factor analyses. The results showed that the items generally loaded onto the expected factors: depression (S1), anxiety (S2), and stress (S3), which together explained 66.1% of the variance; risk perception (F1) and safety attitude (F2), which explained 72.76% of the variance; and safety behavior (SB), which accounted for 76.42% of the variance (see Table 1). Items with factor loadings below 0.60 were excluded, specifically items 19 and 26. The Cronbach’s alpha values for all three factors exceeded 0.6, indicating acceptable internal consistency. The factor analysis results are summarized in Table 2.

### 3.1. Correlation Analysis of PSs, Safety Climate, and Safety Behaviors

The results of the correlation analysis (refer to Table 3) indicate that depression (S1) is significantly correlated with the other two psychological symptoms (i.e., anxiety (S2: 0.572) and stress (S3: 0.557)), two components of safety climate (i.e., risk perception (F1: 0.369) and safety attitude (F2: −0.236)), and two types of safety behaviors (i.e., safety compliance (SB2: −0.249) and safety participation (SB3: −0.246)). Anxiety (S2) is significantly correlated with risk perception (F1: 0.559), risky behavior (SB1: 0.342), safety compliance (SB2: −0.340), and stress (S3: 0.525). Stress (S3) is significantly correlated with risk perception (F1: 0.342) and safety compliance (SB2: −0.460). In terms of safety climate, risk perception (F1) is significantly correlated with two safety behaviors: risky behavior (SB1: 0.374) and safety compliance (SB2: −0.342); safety attitude (F2) is significantly correlated with safety compliance (SB2: 0.326) and safety participation (SB3: 0.233).

### 3.2. Multiple Regression Analysis of PSs, Safety Climate, and Safety Behaviors

All psychological symptoms (PSs) and safety climate variables were selected as independent variables in the multiple regression analysis to investigate the linear relationships among the three types of PSs, safety climate, and safety behaviors. The results are presented in Table 4, which outlines three regression models (Models 1, 2, and 3) to explain the relationships between these variables.

Model 1 reveals that risky behavior (SB1) is positively associated with anxiety (S2) and risk perception (F1), explaining 16.5% of the variance. In Model 2, safety compliance (SB2) is positively associated with depression (S1) and safety attitude (F2), while negatively associated with stress (S3) and risk perception (F1), explaining 36.8% of the variance. Safety participation (SB3) in Model 3 is positively associated with safety attitude (F2) and depression (S1), explaining 9.3% of the variance.

Additionally, regression analyses were conducted to examine the linear relationships between PSs and safety climate. Model 4 shows that safety attitude (F2) is negatively associated with depression (S1) but positively associated with stress (S3), explaining 9.2% of the variance. In Model 5, risk perception (F1) is positively associated with anxiety (S2), explaining 31.3% of the variance. Models 6 to 8 demonstrate that the PSs are all positively associated with each other, with depression, anxiety, and stress explaining 41.8%, 38.9%, and 37.3% of the variance, respectively.

**Figure 2:**
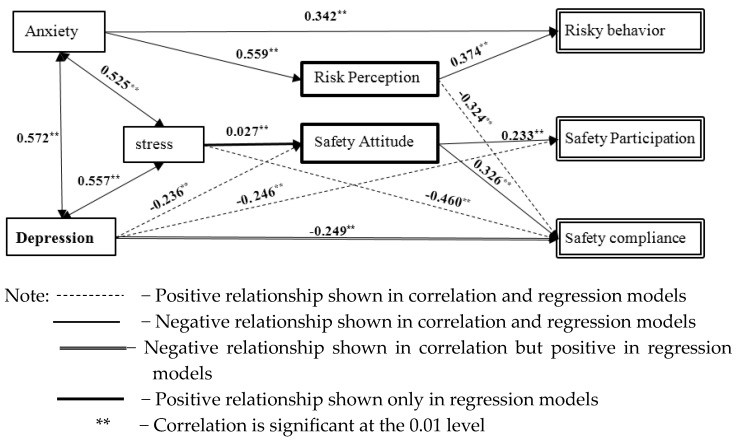
Interrelationships between PSs, safety climate, and safety behaviors of CWs.

The findings from the correlation and regression analyses were synthesized into a ”Psychological Symptoms–Safety Climate–Safety Behavior” model for construction workers (CWs), as illustrated in Figure 2. This model reveals a complex interplay between psychological symptoms (PSs), safety climate, and safety behaviors, offering valuable insights into how psychological factors influence safety outcomes. Specifically, the model suggests that risky behavior is influenced by depression and risk perception, safety participation is driven by safety attitude and depression, and safety compliance is affected by depression, stress, risk perception, and safety attitude. Moreover, the model highlights that depression and stress influence safety attitudes, while anxiety significantly impacts risk perception. An interesting aspect of the model is that each psychological symptom (PS) factor can exacerbate the other two, forming a potentially vicious cycle that further undermines workers’ well-being and safety outcomes.

## 4. Discussion

### 4.1. The Relationship Between Psychological Symptoms and Risky Behavior

Prolonged exposure to adverse working conditions, such as repetitive handling of heavy materials and the use of vibrating tools, often predisposes CWs to anxiety (Kamardeen & Hasan, 2024). Such environmental factors, coupled with high job demands, can lead to increased vulnerability to mental health issues (Bakker & de Vries, 2021). In response to these pressures, CWs frequently resort to coping mechanisms, including smoking, drinking, or gambling, which may provide short-term relief but ultimately worsen long-term psychological health (Sawicki & Szóstak, 2020; Yang et al., 2021). This finding is well-aligned with previous studies that showed anxious individual tend to take more risks (Bhandari et al., 2021; Filbeck et al., 2005).

CWs who perceive risks as unavoidable or beyond their control are less likely to actively engage in safety practices (Zinn, 2019). This perception of risk as a fixed and uncontrollable factor often leads to resignation, diminishing the worker’s willingness to take preventive actions (Moyo et al., 2022). Such workers may view safety measures as futile, which is detrimental to overall safety performance. This mindset reinforces the critical role of individual risk perception in influencing risky behaviors. Supporting prior research (Man et al., 2024), this finding underscores the importance of cultivating a more adaptive and proactive risk perception in workers, which could lead to more effective safety behavior and better risk management strategies on-site.

### 4.2. Psychological Symptoms and Safety Climate as Predictors of Safety Compliance

Psychological symptoms (PSs) play a significant role in shaping safety compliance behaviors. Intriguingly, depression is positively associated with safety compliance which is not consistent with a previous study (Amoako et al., 2023), possibly because depressed CWs, being less proactive or motivated, may avoid rule violations in a passive way (Zheng et al., 2021). This could be a form of emotional withdrawal, where workers are less likely to take risks or deviate from established safety protocols simply because they lack the energy or drive to act otherwise (Chen & Li, 2020). According to Maslow’s hierarchy of needs, safety is a fundamental human necessity (Rojas et al., 2023). Even in a state of depression, CWs may prioritize compliance with safety protocols as a means of fulfilling their basic need for security, thereby reducing the risk of harm to themselves.

Conversely, stress negatively affects safety compliance. CWs experiencing heightened stress may overreact to certain situations, exhibit difficulty concentrating, or experience mental fatigue, all of which hinder their ability to comply with safety regulations. Stress can lead to cognitive overload, making it challenging for workers to pay attention to safety details, ultimately undermining safety compliance (Leung et al., 2016; Misra et al., 2020). This finding aligns with previous studies, such as that of Hemaid Alsulami et al. (Alsulami et al., 2021), which demonstrated that workers’ stress levels in Saudi Arabia negatively impacted their safety compliance. Similarly, CWs with a low perception of risk are less likely to comply with safety practices, as they perceive risk-taking as a normal aspect of their work, rather than something to avoid (Danso et al., 2023). A lower risk perception can contribute to non-compliance by making workers underestimate potential hazards. This is consistent with the study by Omidi et al. (2023), which found that safety compliance is negatively predicted by risk perception. On the other hand, a positive safety attitude fosters greater compliance, as workers who view safety as a priority are more likely to engage in behaviors that protect themselves and others from harm (Liu et al., 2019; Saleem et al., 2022).

### 4.3. Depression and Safety Attitude as Predictors of Safety Participation

Safety participation behaviors, which encompass proactive efforts to enhance workplace safety, are strongly influenced by both depression and safety attitude. Depressed CWs often struggle to engage in activities such as reporting hazards (Chan et al., 2020), suggesting safety improvements, or participating in safety-related training or discussions. This lack of engagement could stem from a sense of apathy or emotional exhaustion, which hinders their capacity for active involvement in safety initiatives (Chen et al., 2017). As consistently highlighted in a previous study, workers experiencing depression often exhibit a lack of commitment to their jobs due to negative emotions (Zhang et al., 2022), which further reduces their commitment to safety participation. In contrast, workers with a positive safety attitude tend to exhibit higher levels of safety participation, which is consistent with a previous study (Saedi et al., 2020). These individuals prioritize safety, not only in terms of compliance but also by actively contributing to creating a safer work environment through proactive behaviors, such as suggesting improvements or addressing safety concerns before they lead to accidents.

### 4.4. Psychological Symptoms as Predictors of Safety Climate

Anxiety can severely detriment safety climate by impairing CWs’ ability to concentrate and make sound decisions. Previous studies have also indicated a strong association between anxiety and risk perception (Butler & Mathews, 1987; Oyetunji et al., 2023). Anxious CWs may rush through tasks in an attempt to alleviate their distress, leading to overlooking of safety measures and increased risk-taking behaviors (Tan, 2022). This finding emphasizes the importance of managing anxiety, not just as a mental health issue, but also as a factor that can undermine safety culture in the workplace. On the other hand, stress can have a positive influence on safety attitude, particularly among experienced CWs who have witnessed or been directly affected by on-site accidents (Liang et al., 2022). Such experiences often lead to a more cautious and safety-conscious mindset, as workers become more aware of the potential dangers in their environment (Saleem & Malik, 2022). However, depression generally has a negative impact on safety attitude, as workers with depression often find it difficult to focus on safety matters, due to the overwhelming nature of their negative emotions. This is confirmed by previous research, which revealed a significant association between workers’ depression and safety attitude (Denning et al., 2021).

### 4.5. Anxiety as a Precursor to Depression

Intensive, repetitive tasks in hazardous environments frequently induce anxiety in CWs. This anxiety manifests as physical symptoms such as trembling, respiratory distress, or elevated heart rate, all of which can negatively impact work performance. Anxiety can cause workers to become overly focused on their distress, leading them to neglect critical safety measures or take unnecessary risks to alleviate their discomfort (Brosschot et al., 2016). Over time, sustained anxiety can lead to burnout and psychological exhaustion, culminating in depression (Bianchi et al., 2018). This aligns with extensive research across various domains that establishes a robust link between anxiety and depression (Lim et al., 2017). The progression from anxiety to depression suggests a feedback loop, where the persistent presence of anxiety eventually leads to emotional depletion, which in turn exacerbates the underlying mental health condition and further compromises safety behaviors.

## 5. Implications

The findings of this study offer practical insights into managing psychological symptoms (PSs) to enhance construction workers’ (CWs) safety behaviors. The results highlight that risky behavior is influenced by depression and risk perception, while safety compliance is negatively impacted by stress and risk perception. A positive safety attitude significantly promotes safety participation and compliance. Moreover, risk perception predicts risky behavior and inversely affects safety compliance. Depression, while negatively impacting safety attitude and participation, is paradoxically associated with higher safety compliance. Anxiety emerges as a precursor to risk perception, risky behavior, and stress.

### 5.1. Practical Implications

The findings of this study offer valuable insights for improving safety management in the construction industry. First, the significant influence of psychological symptoms (e.g., depression, stress, and anxiety) on construction workers’ safety behaviors highlights the need for companies to prioritize mental health. Providing psychological counseling, stress management training, and mental health support programs can help mitigate the adverse effects of psychological symptoms, thereby enhancing overall safety performance. Considering that many construction workers in China live apart from their families for extended periods, this separation often exacerbates psychological symptoms. To address this, construction contractors should consider measures such as facilitating regular video calls with family members (Kelly et al., 2023), offering travel reimbursements (Ihemereze et al., 2023), or providing monthly opportunities for workers with good safety performance to visit their families, thereby strengthening social bonds and well-being.

Second, the study underscores the critical role of a positive safety climate in moderating the relationship between psychological symptoms and safety behaviors. Enterprises should foster such a climate by enhancing safety training, clarifying safety responsibilities, and promoting open safety communication. Regular safety meetings can also improve the exchange of safety-related information between workers and management (Maliha et al., 2021).

Furthermore, the study highlights that different safety behaviors are influenced by distinct psychological factors. For instance, risky behaviors are primarily shaped by depression and risk perception, safety participation is driven by safety attitudes and depression, while safety adherence is affected by depression, stress, risk perception, and safety attitudes. Regular mental health training should be offered to construction workers to help them recognize early signs of psychological distress and equip them with coping strategies for managing stress effectively (Zhao, 2021).

Lastly, the mutual exacerbation of psychological symptoms suggests the potential for a vicious cycle of mental health problems. Early identification and intervention are essential to prevent the accumulation and worsening of these symptoms, ultimately safeguarding workers’ well-being and safety performance. Contractors could collaborate with university researchers to develop a user-friendly app specifically designed for construction workers to monitor psychological symptoms. This app could prompt workers to complete a weekly self-assessment, with a particular focus on identifying those with poor psychological symptoms, enabling targeted interventions for those in need (Fiol-DeRoque et al., 2021).

### 5.2. Theoretical Implications

This study also makes notable theoretical contributions. The proposed “psychological symptoms–safety climate–safety behavior” model introduces a novel framework for understanding the impact of psychological factors on safety behaviors. This model not only identifies the direct effects of psychological symptoms on safety behaviors but also highlights their indirect influence through safety climate, enriching the theoretical foundations of safety behavior research.

Additionally, the study reveals that psychological symptoms such as depression, stress, and anxiety exacerbate one another, forming a vicious cycle. This finding offers new theoretical support for mental health research by emphasizing the dynamic and interconnected nature of psychological symptoms, paving the way for future investigations into their cumulative effects.

The study further establishes the moderating role of safety climate between psychological symptoms and safety behaviors, broadening the conceptual understanding of safety climate. This finding suggests that safety climate serves not only as a precursor to safety behaviors but also as a key mediating variable between psychological factors and safety behaviors.

Moreover, by distinguishing between risky behavior, safety participation, and safety compliance, and identifying their unique psychological drivers, the study provides a theoretical basis for classification-based research on safety behaviors. Finally, the integration of psychological and safety management theories advances interdisciplinary research, offering a comprehensive mechanism for understanding how psychological factors influence safety behaviors and suggesting new directions for future studies.

In summary, this study provides actionable recommendations for enhancing safety management in the construction industry while making significant contributions to the theoretical development of safety behavior research and related fields.

## 6. Conclusions

This study underscores the critical role of psychological symptoms (PSs) in shaping safety climate and safety behaviors among construction workers (CWs). Risky behavior was found to be driven by workers’ anxiety and risk perception, while safety participation was positively associated with safety attitudes. Safety compliance, however, was negatively influenced by stress, risk perception, and depression. Notably, depression not only contributed to poor safety attitudes but also exhibited a counterintuitive inverse relationship with risky behavior, highlighting the complexity of its effects on safety outcomes.

Building on these findings, this study offers several actionable recommendations aimed at mitigating psychological symptoms and promoting safer behaviors in construction settings. Practical strategies include facilitating regular video communication with family members to alleviate loneliness, offering travel reimbursements, and providing monthly opportunities for high-performing workers to visit their families. Additionally, ensuring access to comprehensive medical and psychological support services can address underlying mental health challenges. These insights contribute to a deeper understanding of the interplay between psychological factors and safety behaviors, particularly in the context of China’s construction industry. The proposed strategies not only provide site managers with a framework for enhancing worker well-being but also serve as a foundation for future research to validate and adapt these findings in diverse cultural and industrial settings.

Despite the significant findings of this study, several limitations should be noted. First, the data for this study were collected primarily from construction sites in Shandong and Jiangsu provinces, two of China’s most active regions in the construction industry. The majority of the respondents were migrant workers, many of whom came from other provinces such as Sichuan, Henan, and Jiangxi. Although this study focused on Shandong and Jiangsu, the inclusion of construction sites from other provinces provides a broader perspective on the psychological challenges faced by migrant construction workers. Future studies could expand the sample to include workers from additional regions to further explore the geographical and social factors that may impact their mental health and safety behaviors.

Second, although this study was conducted in Mainland China, its findings provide insights that may be relevant to other countries with similar construction industry conditions, such as high manual labor, challenging working environments, and limited worker mobility. Psychological distress among construction workers, including depression, anxiety, and stress, is a global issue, and the safety climate factors identified here, such as risk perception and safety attitude, are likely applicable internationally. However, cultural and environmental differences should be considered when applying these results to other contexts. Future research could examine how psychological factors interact with safety behaviors across diverse cultures to improve understanding of worker well-being in different construction settings.

Third, this study focuses specifically on the interplay between psychological stress (stress, anxiety, and depression), safety climate, and safety behaviors among construction workers. While psychological disorders, such as mania, are recognized as significant issues in some contexts, this research is not designed to diagnose or analyze specific psychological disorders. Instead, it aims to explore general psychological symptoms and their influence on workplace safety behaviors. Future studies could delve into the impact of psychological disorders on construction workers’ safety outcomes to provide a more comprehensive understanding.

Fourth, the sample for this study may not fully reflect the broader industry’s age distribution, particularly in the 45–55 age group. The majority of our respondents were aged 30–39 (36.8%), with a significant portion in the 40–49 group (31.6%). Future research could aim for a more representative age distribution across different regions to further explore how psychological symptoms and safety behaviors may vary with age. Lastly, this study primarily concentrated on examining the influence of psychological pressures on safety climate and safety behaviors, leaving other critical factors—such as family-related factors, organizational leadership styles, worker support, and leadership support—for future investigation.

## Figures and Tables

**Table 1:** Overview of variables and corresponding references used in this study.

Items	Variables	References
1	Depression	(Moussa et al., 2017)
2	Anxiety	(Moussa et al., 2017)
3	Stress	(Moussa et al., 2017)
4	Risk perception	(Siegrist & Árvai, 2020)
5	Safety attitude	(Oah et al., 2018)
6	Risky behavior	(Han et al., 2022).
7	Safety compliance	(Hu et al., 2020)
8	Safety participation	(Choi & Lee, 2022)

**Table 2:** Scale items, factor loading, and coefficient alpha for the psychological symptoms, safety climate, and safety behavior of CWs.

Factors		Items	Description	Factor Loading	Alpha
Psychological symptoms					
S1	Depression	1.	I felt down-hearted and blue	0.797	0.853
		2.	I felt that I had nothing to look forward to	0.772	
		3.	I was unable to become enthusiastic about anything	0.669	
		4.	I found it difficult to work up the initiative to do things	0.645	
S2	Anxiety	5.	I was aware of the action of my heart in the absence of physical exertion (e.g., sense of heart rate increase, heart missing a beat)	0.809	0.824
		6.	I experienced trembling (egg, in the hands)	0.799	
		7.	I experienced breathing difficulty (e.g., excessive breathing, breathlessness in the absence of exertion)	0.683	
		8.	I felt I was close to panic	0.662	
S3	Stress	9.	I felt that I was rather touchy	0.818	0.847
		10.	I was intolerant of anything that kept me from getting on with what I was doing	0.817	
		11.	I found myself getting upset by quite trivial things	0.712	
		12.	I tended to over-react to situations	0.675	
		13.	I found it difficult to relax	0.620	
Safety climate					
F1	Risk perception	14.	Risk or not is decided by the gods, I could do nothing about it.	0.808	0.701
		15.	Risk-taking is part of my job	0.787	
		16.	I think it is very likely for me to be injured on the job in the next 12-month period	0.767	
F2	Safety attitude	17.	My own safety on site is very important	0.862	0.613
		18.	If my supervisor is careful about safety on site, that makes me careful too.	0.793	
		19 *.	If my workmates care less about safety on site, that makes me careless too.	0.230	
Safety behavior					
SB1	Risky behavior	20.	I always get drunk and smoke a lot.	0.878	0.823
		21.	I like gambling, and I usually play at least one kinds of the following gambling: cards, mahjong, slot machine, or other gambling styles.	0.854	
		22.	I often engage in high-speed operation of construction vehicles or machinery on-site.	0.827	
SB2	Safety compliance	23.	I use all the necessary safety equipment to do my job.	0.929	0.896
		24.	I use the correct safety procedures for carrying out my job.	0.906	
		25.	I work according to a safe work-pace (no rush)	0.827	
		26 *.	I usually use of appropriate and non-defective tools and equipment	0.398	
SB3	Safety participation	27.	I voluntarily carry out tasks or activities that help to improve workplace safety.	0.844	0.863
		28.	I always not only keep myself safe, but remind workmates to work safely.	0.838	
		29.	I proactively report safety hazards.	0.811	
		30.	I put in extra effort to improve the safety of the workplace.	0.789	

Note: *—Items 19 and 26 were removed due to their low factor loadings.

**Table 3:** Correlation between PSs, safety climate, and safety behaviors.

Factors	Psychological Symptoms			Safety Climate		Safety Behaviors		
	S1	S2	S3	F1	F2	SB 1	SB 2	SB 3
S1—Depression	1.000 **							
S2—Anxiety	0.572 **	1.000 **						
S3—Stress	0.557 **	0.525 **	1.000 **					
F1—Risk perception	0.369 **	0.559 **	0.342 **	1.000 **				
F2—Safety attitude	−0.236 **	−0.113	0.027	−0.096	1.000 **			
SB1—Risky behavior	0.130	0.342 **	0.158 *	0.374 **	0.053	1.000 **		
SB2—Safety compliance	−0.249 **	−0.340 **	−0.460 **	−0.324 **	0.326 **	−0.017	1.000 **	
SB3—Safety participation	−0.246 **	−0.083	−0.122	−0.183 *	0.233 **	−0.211 **	0.381 **	1.000 **

Note: **—Correlation is significant at the 0.01 level (2-tailed). *—Correlation is significant at the 0.05 level (2-tailed).

**Table 4:** Regression models for PSs, safety climate, and safety behaviors.

		Model	B	Std. Error	t	Sig.	VIF	R	R2	Significance (ANOVA)
	Risky behavior ← PSs, Safety climate									
1.		(Constant)	1.556	0.409	3.806	0.000		0.407	0.165	0.000
	F1	Risk Perception	0.347	0.115	3.015	0.003	1.455			
	S2	Anxiety	0.252	0.114	2.202	0.029	1.455			
	Safety compliance ← PSs, Safety climate									
2.		(Constant)	4.901	0.541	9.053	0.000		0.607	0.368	0.000
	S3	Stress	−0.595	0.092	−6.449	0.000	1.559			
	F3	Safety Attitude	0.373	0.068	5.481	0.000	1.104			
	F1	Risk Perception	−0.212	0.081	−2.620	0.010	1.198			
	S1	Depression	0.238	0.102	2.329	0.021	1.665			
	Safety Participation ← PSs, Safety climate									
3.		(Constant)	4.622	0.540	8.556	0.002		0.305	0.093	0.000
	S1	Depression	−0.222	0.086	−2.589	0.011	1.059			
	F3	Safety Attitude	0.166	0.070	2.369	0.019	1.059			
	Safety attitude ← PSs									
4.		(Constant)	5.548	0.415	13.368	0.000		0.303	0.092	0.001
	S1	Depression	−0.446	0.112	−3.972	0.000	1.449			
	S3	Stress	0.262	0.104	2.510	0.013	1.449			
	Risk perception ← PSs									
5.		(Constant)	1.679	0.249	6.737	0.000		0.559	0.313	0.000
	S3	Anxiety	0.556	0.066	8.482	0.000				
	Depression ← Stress, anxiety									
6.		(Constant)	1.377	0.252	5.455	0.000		0.647	0.418	0.000
	S2	Anxiety	0.357	0.066	5.401	0.000	1.380			
	S3	Stress	0.329	0.067	4.945	0.000	1.380			
	Anxiety ← Depression, stress									
7.		(Constant)	0.671	0.300	2.234	0.027		0.624	0.389	0.000
	S1	Depression	0.438	0.081	5.401	0.000	1.449			
	S3	Stress	0.301	0.076	3.985	0.000	1.449			
	Stress ← Depression, anxiety									
8.		(Constant)	1.175	0.292	4.021	0.000		0.611	0.373	0.000
	S1	Depression	0.409	0.083	4.945	0.003	1.487			
	S2	Anxiety	0.305	0.077	3.985	0.000	1.487			

## Data Availability

The original contributions presented in the study are included in the article, further inquiries can be directed to the corresponding authors.
